# Mechanical Properties and Axial Compression Deformation Property of Steel Fiber Reinforced Self-Compacting Concrete Containing High Level Fly Ash

**DOI:** 10.3390/ma15093137

**Published:** 2022-04-26

**Authors:** Pan Liu, Ran Hai, Junxia Liu, Zhiquan Huang

**Affiliations:** 1 College of Geosciences and Engineering, North China University of Water Resources and Electric Power, Zhengzhou 450046, China; liupan201498@126.com; 2 School of Architectural Engineering, Zhongyuan University of Technology, Zhengzhou 450007, China; liujunxia80600@163.com; 3 Luoyang Institute of Science and Technology, Luoyang 471023, China

**Keywords:** self-compacting concrete, fly ash, steel fibers, mechanical characteristics, axial compression deformation characteristics

## Abstract

The cement industry has brought serious environmental pollution problems. In the background of ecological civilization, accelerating rational use of waste resources plays an important role in protecting the environment. In this study, self-compacting concrete (SCC) is prepared using fly ash and lime powder as supplementary cementitious materials by replacing 50%, 60%, and 70% of ordinary Portland cement. By systematically analyzing the influence of the fly ash replacement rate on the workability and mechanical properties of SCC, steel-fiber-reinforced SCC containing 60% fly ash is chosen for further study, and steel fiber is added at the percentages of 0.25%, 0.50%, 0.75%, and 1.00%. The performances in fresh and hardened states are investigated in terms of workability, compressive strength, splitting tensile strength, flexural strength, and axial compression deformation property. The obtained outcomes indicate that although the incorporation of fly ash can improve the workability of the mixture, there is a negative correlation between the mechanical properties of SCC and the fly ash replacement rate. For steel-fiber-reinforced SCC containing 60% fly ash, when the content of steel fibers exceeds 0.75%, the workability decreases sharply, and even when the volume fraction is 1.00%, the passing ability cannot meet the requirements of the technical specifications for applications of self-compacting concrete. The analysis results for mechanical properties show that compressive strength is not changed significantly with increasing percentage of steel fibers. The steel fibers strengthen splitting tensile strength and flexural strength significantly, and compared with that of without steel fibers, they increased by 22% and 58%, respectively, with steel fibers up to 1.00%. Additionally, the parameters of the axial compression deformation property are improved by introducing steel fibers, especially the strain energy ($V_{\varepsilon }$) and relative toughness (Γ) of steel-fiber-reinforced SCC containing a high level of fly ash.

## 1. Introduction

SCC is a type of green high-performance concrete, which is widely used in different projects and research due to its high fluidity, uniformity, and stability. It can flow and fill the formwork space under its own weight without external force vibration [1]. Compared with ordinary concrete of the same strength, SCC has characteristics of low water-to-cement ratio [2], large quantity of fine aggregate [3], and large amount of cement and additives [4], thereby resulting in the disadvantages of high hydration heat, high shrinkage, and expensive cost [5]. SCC is prepared using fly ash as a supplementary cementitious material, as fly ash contributes to the fresh SCC’s workability, as well as long-term durability properties [6,7,8].

The fly ash can improve the micro-structure of concrete through the morphological effect, micro-aggregate effect, and pozzolanic effect, further affecting its macro-properties [9,10]. However, the improvement in performance of SCC containing fly ash presents different characteristics. It was reported that the use of fly ash and limestone powder to partially replace cement gives SCC better workability, that is, shorter flow time (T_500_) and greater slump flow of the mixture [6,11]. The study of P. Dinakar et al. [12] found that the slump flow of SCC tends to increase first and then decrease in range of 10–70% fly ash, and when the fly ash is 70%, slump flow drops slightly compared with mixtures containing 50% fly ash, while T_500_ and flow time of a V-shaped funnel do not change significantly with fly ash content. Compared with ordinary Portland cement, the fly ash has a slower pozzolanic reaction, so the compressive strength of SCC with 50–70% fly ash is lower than that of SCC prepared using pure cement for each test age [13]. Similarly, it was observed that the splitting tensile strength and flexural strength is negatively correlated with the fly ash content [14,15]. This suggests that the incorporation of large amounts of fly ash causes SCC to show lower strength, particularly in the early stages, since fly ash cannot make up for the loss of cement [16,17], while the reduction in strength can be improved at the later ages to some extent [18].

Adding steel fibers is the main way to improve the strength and deformation properties of concrete [19], and the propagation of cracks can be effectively inhibited due to the bridging function of steel fibers. Compared to SCC without steel fibers, the compressive strength of SCC with 0.5% and 1.0% steel fiber is increased by 6% and 8%, respectively [20]. The improvement in tensile strength and flexural strength of SCC by adding steel fibers is better than that in compressive strength [21,22]. Different behavior is observed in other literature [23]. A. Alrawashdeh et al. [23] observed that compared to the control mix, the use of steel fibers makes compressive strength worse, but splitting tensile strength is enhanced. Zeyad [24] noted that the decrease of compressive strength mainly depends on the length and shape of steel fibers. On the basis of the above research, although the influence of steel fibers on compressive strength is debatable, it causes concrete to show better ductility and post-peak behavior [25]. By analyzing stress-strain curves of steel-fiber-reinforced concrete, Wang et al. [26] revealed that the addition of steel fibers markedly changes the failure mode of concrete from brittleness to ductility, and the toughness energy is positively correlated with steel fibers content in both static and dynamic compression. A study on steel-fiber-reinforced expanded-shale lightweight aggregate concrete (SFRELC) found that the compressive toughness ratio of SFRELC was 1.61–2.06 times greater than that of all-lightweight-aggregate concrete with steel fibers changed from 0.8% and 2.0% [27]. Yang et al. [28] results show that steel fibers can significantly enhance the tensile-compression ratio and flexural-compression ratio of high-fluidity ultra-high-strength concrete, and its relative toughness under axial compression is twice that of the matrix.

The results above imply that the workability of SCC is improved by partially replacing ordinary Portland cement, but the mechanical properties of SCC are reduced. On the contrary, the introduction of steel fiber can enhance the bending mechanical properties and toughness of concretes, but it’s known to all that the influence of steel fibers on the workability of SCC is particularly unfriendly [29,30]. For this reason, scholars have proposed adding both fly ash and steel fiber into SCC [31], which can not only improve the workability of SCC, but also be beneficial to its mechanical properties and toughness. At present, the research mainly focuses on SCC with small volumes of fly ash (10–20%). In this paper, SCC is prepared with high levels of fly ash/lime powder (50%, 60%, and 70% by weight of cement), and tests of the workability and mechanical properties are carried out. Based on the analysis results of the workability and mechanical properties of SCC with 50–70% fly ash, SCC with 60% fly ash and different volume fractions of steel fiber (0%, 0.25%, 0.50%, 0.75%, and 1.00%) is prepared, and its workability, mechanical properties, and parameters of axial compression deformation characteristics are discussed. This is expected to provide a reference for further research and applications of steel-fiber-reinforced SCC with high levels of fly ash.

## 2. Materials and Methods

### 2.1. Raw Materials

Ordinary Portland cement with a grade of 42.5 was used in light of Chinese standard specifications (GB175-2007) [32]. The properties of ordinary Portland cement are listed in Table 1. The 45 μm sieving residue of them is 2.55%, which is determined by vacuum sieving method. The water requirement for normal consistency of Portland cement is 27.3%. Its specific surface area is 358 m^2^/kg, density is 3.03 g/cm^3^, and fluidity of cement mortar is 192 mm.

Fly ash has a specific surface area of 463 m^2^/kg, water content of 0.18%, water requirement ratio of 104.32%, and 28 d activity index of 79.34%. The main components of fly ash are presented in Table 2.

Lime powder produced by Liannanqiao building materials store, Shaping Town, Heshan City (in Guangdong, China) was used in this study.

Mixed sand with a fineness modulus of 2.75 was employed as fine aggregate, and apparent density and bulk density are 2.65 g/cm^3^ and 1.52 g/cm^3^, respectively.

Crushed limestone with 5–19 mm continuous gradation was employed as coarse aggregate. The bulk density and apparent density are 1.56 g/cm^3^ and 2.61 g/cm^3^, respectively.

Tap water was used in this study.

A polycarboxylate-based water-reducing agent of 530P type was used in this study, supplied by Nanjing Sitaibao Trading Co. Ltd. (in Nanjing, Jiangsu, China), with a water reduction rate of 30%.

**Figure 1:**
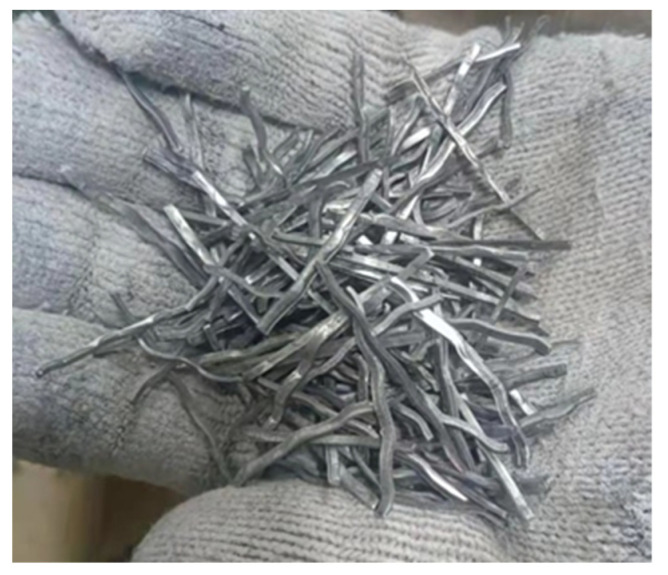
Steel fibers.

Corrugated steel fiber produced by Hengshui Ruihai Rubber Products Co. Ltd. (in Hengshui, Hebei, China) was used in this study. The picture and property parameters of steel fibers used in this study are given in Figure 1 and Table 3, respectively.

### 2.2. Mix Proportions

The performance of SCC is controlled by the raw materials and mix proportion. In this paper, SCC is made with fly ash/lime powder instead of 50%, 60%, and 70% (by weight) of the cement. The addition of lime powder is to improve the workability of SCC [33]. See Table 4 below for the mix proportions of mixture. By systematically analyzing the workability and mechanical properties of SCC containing 50%, 60%, and 70% fly ash, steel-fiber-reinforced SCC containing 60% fly ash along with steel fiber (volume fractions of 0%, 0.25%, 0.50%, 0.75%, and 1.00%) is selected for further study.

### 2.3. Experimental Methods

A horizontal mixer was used to mix materials weighed according to their mix proportions in advance. First, coarse aggregate, fine aggregate, and cementitious materials (cement, fly ash, and lime powder) were added the mixer, and dry mixed for about 60 s. Second, half of the water dissolved in water-reducing agent was added and mixed for another 60 s. Thirdly, steel fibers were evenly sprinkled into the mixture within another 90 s during the process of mixing (when the volume fraction of steel fiber was 0%, the mixture was stirred for 90 s without steel fiber). At last, the remaining half of the water was added into the mixture and mixed for about 90 s until a homogeneous mixture was obtained. All mixtures were mixed in this way.

**Figure 2:**
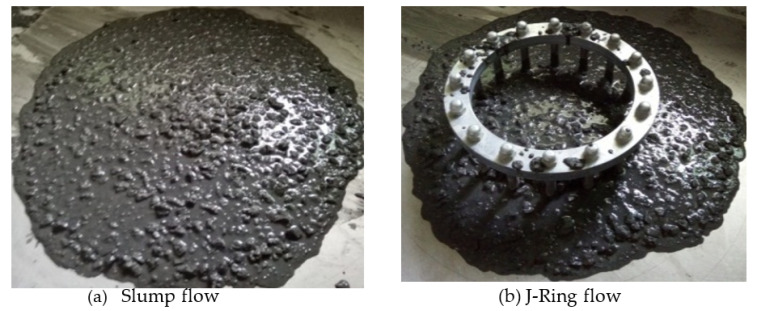
Workability testing.

The workability test is performed, including the slump flow, slump-flow time (T_500_), J-Ring flow, and static segregation percent. The slump flow is characterized by the average value of the maximum diameter in two vertical directions after the mixture stops flowing. Slump-flow time (T_500_) is the time when the slump flow reaches 500 mm after releasing the slump cylinder. J-Ring flow refers to the average value of the maximum diameter in two vertical directions when mixture flows through a steel crown after releasing the slump cylinder. Segregation rate is represented by static segregation percent, which is the ratio of the mass of mortar flowing through a standard sieve to that of the concrete after the concrete has been standing for 120 s ± 5 s in the sieving experiment. All tests were carried out in accordance with the Chinese specification JGJ/T 283-2012 [34]. Figure 2 shows part of the workability test process.

**Figure 3:**
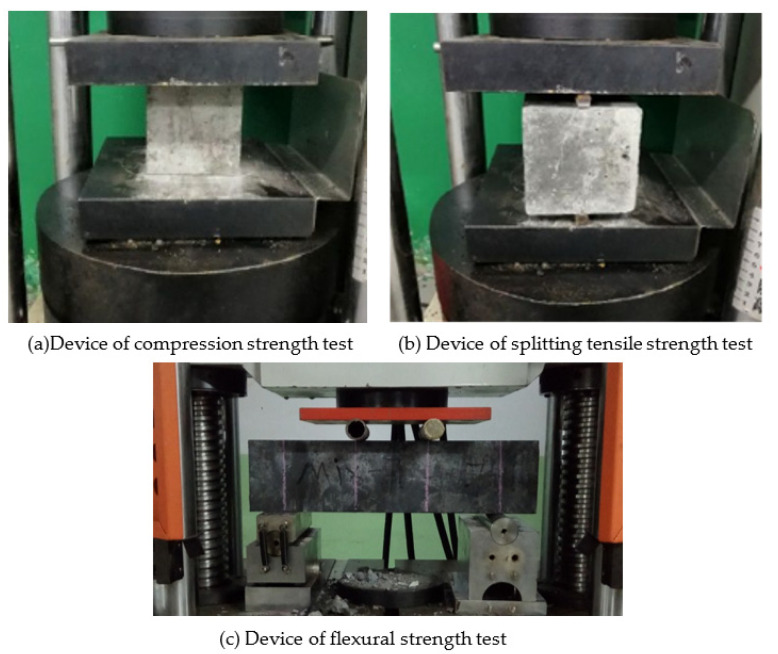
Device used for mechanical properties test.

Mechanical properties of hardened SCC include compression, splitting tensile, and flexural strength. Cubical specimens are used for tests of compressive strength and splitting tensile strength. The size of the specimens is 100 mm × 100 mm × 100 mm. The size of the flexural strength specimen is 100 mm × 100 mm × 400 mm. Three specimens were measured at the age of 28 d for each case, and the average results were reported. Figure 3 shows the device used for the mechanical properties test.

**Figure 4:**
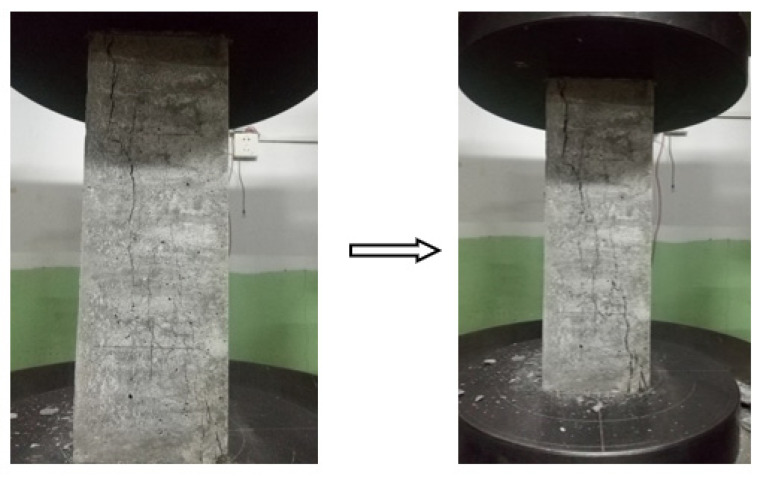
Crack propagation process of specimen in axial compression deformation test.

The axial compression deformation performance test was carried out at 28 d age using the YAW6206 electro-hydraulic servo-pressure-testing machine-controlled microcomputer. The size of the specimen was 100 mm × 100 mm × 300 mm. The loading mode was constant displacement, and the loading speed was 0.05 mm/min. The surface crack propagation process of the specimen during the test is shown in Figure 4.

## 3. Results and Discussion

### 3.1. Properties of SCC with Different Fly Ash Content

#### 3.1.1. Workability

The results of workability are displayed in Table 5. The test criteria of filling ability are determined by slump flow and T_500_ together. The passing ability is decided by the D-value between slump flow and J-Ring flow, and segregation resistance is determined by the separation rate. The utilization of fly ash to substitute for cement makes SCC exhibit greater slump flow and shorter flow time (T_500_), which shows that the filling ability of SCC is ameliorated. Moreover, as the replacement rate increases from 50% to 70%, the filling ability of the mixture is improved more remarkably. In terms of passing ability, all test results meet the specification requirements: not more 50 mm (JGJ/T 283-2012) [34]. Whereas there is a fluctuating change in passing ability, the total fluctuation range is not obvious with the D-value of 5 mm yet. Generally speaking, when the replacement ratio is 60%, the passing ability of SCC is better. Besides that, the separation rate gradually decreases from 15.3% to 11.6% when the replacement rate is increased from 50% to 70%, indicating that the mixture has better segregation resistance performance with the increase of fly ash content.

The influence mechanism of fly ash on the workability of SCC may be due to the following two aspects: (1) the apparent density of fly ash is lower compared to ordinary Portland cement. When cement is substituted for fly ash, it may give rise to an increase in the total amount of cementitious material. Under the joint action of the spherical particle morphology of fly ash, the frictional resistance between aggregate particles in the fresh state decreases significantly, thus improving the working performance of SCC. (2) The fly ash can displace filling water in mortar pores due to its own small particles during early cement hydration, and further release more free water to a certain extent, which increases the fluidity of the mixture and improves the workability of the SCC.

#### 3.1.2. Mechanical Properties

**Figure 5:**
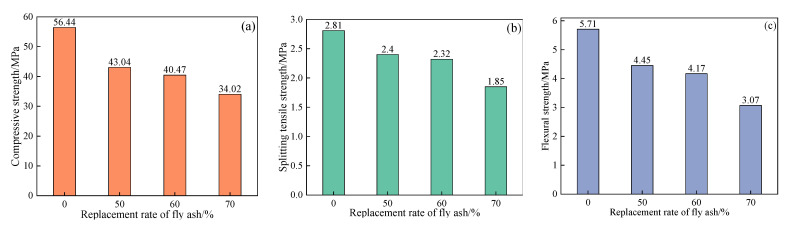
The mechanical properties of SCC with high level fly ash at 28 d age: (**a**) compressive strength; (**b**) splitting tensile strength; (**c**) flexural strength.

Figure 5 presents the trial results of mechanical properties of SCC with 50–70% of fly ash at 28 d age. It is clear that there is a negative correlation between mechanical properties of SCC and replacement rate of fly ash. Similar behavior was observed in literature about high-volume fly ash concrete [16]. Interestingly, when the replacement rate increases from 60% to 70%, the reduction range of the mechanical properties becomes more obvious. Compared to specimens without fly ash, the compressive strength, splitting tensile strength, and flexural strength with 70% fly ash are reduced by 39.72%, 34.16%, and 46.23%, respectively.

The reason may be that when cement is partially substituted with fly ash, the reduction of cement content in SCC results in lower amounts of the C-S-H and CH content produced by the cement hydration reaction. Moreover, many unreacted fly ash particles remain in the matrix. The combined action leads to deterioration in the pore structure of the matrix [35]. In other words, due to an increase in fly ash percentage, the pozzolanic effect of fly ash cannot give full play to their advantages due to low CH amount from the cement hydration process, which reduces the strength of SCC.

### 3.2. Properties of Steel-Fiber-Reinforced SCC Containing High Level of Fly Ash

In view of the above analysis of workability and mechanical properties of SCC containing high levels of fly ash, steel-fiber-reinforced SCC with 60% fly ash is prepared to discuss its workability, mechanical properties, and axial compression deformation characteristics.

#### 3.2.1. Workability

The experimental results regarding the workability of the fresh mixture with steel fibers can be found in Table 6. These data show that slump flow decreases and T_500_ time is prolonged as the volume fraction of steel fibers grows, showing that the increase of steel fibers reduces the filling ability of SCC with a high level of fly ash. After the introduction of steel fiber, the D-value between slump flow and J-Ring flow becomes increasingly prominent, meaning that the passing ability of the mixture becomes worse. The D-value also increases sharply for steel fiber contents exceeding 0.75%, which cannot even meet the Chinese specification requirements (between 0–50 mm) (JGJ/T 283-2012) [34]. This indicates that there is an optimum critical value for steel fibers in passing ability. There is a negative correlation between segregation rate and steel fibers volume fraction, suggesting that introduction of steel fibers helps to improve the segregation resistance of the mixture.

The influencing factors of steel fibers volume fraction on the workability of SCC containing a high level of fly ash are explained by the following two aspects. First, with the introduction of steel fiber into the fresh concrete, there are more steel fiber surfaces, requiring more cement paste. That is to say, as the volume fraction of steel fibers increases, the quantity of cement paste used to encapsulate aggregates and steel fibers in the mixture system increases, thereby leading to a decrease of the free mortar content in the mixture. Therefore, the friction resistance is increased when the mixture flows. Secondly, when increasing the amount of steel fibers distributed randomly in the three-dimensional spatial structure of concrete, the mutual overlap of them becomes more remarkable. Then, a spatial network structure is formed in the SCC, resulting in the cement paste being agglomerated in the network structure. Thus, the filling ability and passing ability of the mixture are hindered.

#### 3.2.2. Mechanical Properties

**Figure 6:**
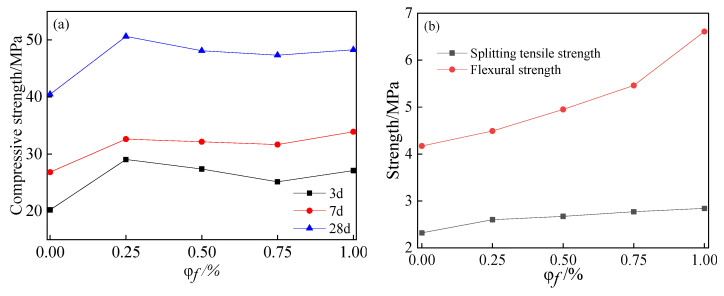
Influence of steel fiber volume fraction ($\varphi_{f}$) on mechanical properties of SCC containing 60% fly ash: (**a**) compressive strength; (**b**) splitting tensile strength and flexural strength.

Figure 6 shows the strength outcomes of steel-fiber-reinforced SCC containing 60% fly ash. The results from Figure 6a show that the compressive strength at each age is higher than that without steel fibers. The compressive strength of SCC changes significantly at the early age along with the range of steel fibers, from 0.25% to 1.00%. However, the variation range of compressive strength gradually decreases with age, and the change range is only 2–5% at 28 d age. This illustrates that the inclusion of steel fibers is beneficial to the compressive strength of SCC containing a high level of fly ash, but the improvement is not always increased with a larger percentage of steel fibers. This result shows no difference with the research by Gencel et al. [36].

This may depend on the fact that steel fibers dispersed in concrete can share the load borne by the cement paste during the loading process. At this time, the crack resistance of steel fibers plays a dominant role; thus, its compressive strength is improved. However, the introduction of steel fibers will also bring a large number of new mortar–steel fiber interfaces, and the bond strength of the interface is relatively low. There is an increasing number of low-strength mortar–steel fiber interfaces with a larger content of steel fibers, further causing the relative reduction in compressive strength. The failure of steel-fiber-reinforced concrete is usually impacted by the combined action of the low-strength matrix–steel fiber interface and the crack resistance of steel fibers. Both of these offer respective advantages in the process of compression and jointly decide the compressive strength of concrete, ultimately resulting in the change of the steel fibers having no obvious influence on the compressive strength of SCC containing a high level of fly ash.

There is a different trend in splitting tensile strength and flexural strength. As shown in Figure 6b, the splitting tensile strength and flexural strength increase with the steel fibers volume fraction. The research of R. Madandoust et al. [37] presents similar results, showing that when the volume fraction of steel fibers is 1.00%, the splitting tensile strength and flexural strength of SCC are 2.84 MPa and 6.61 MPa, respectively, which are 22% and 58% higher than that of SCC without steel fibers. This emphasizes that the splitting tensile strength and flexural strength are improved due to the bridging of cracks by steel fibers. The incorporation of steel fibers controls the propagation of matrix cracks. Steel fiber dispersed in concrete bears tensile stress, and the propagation speed of matrix cracks is delayed during loading, which means the concrete is not damaged until the steel fiber is broken or pulled out from matrix. It is observed that specimens without steel fiber display brittle fracture, while after the incorporation of steel fibers, the fracture surface of the specimen is connected with steel fiber and not completely disconnected, showing ductility in the experiment.

#### 3.2.3. Axial Compression Deformation Property

**Figure 7:**
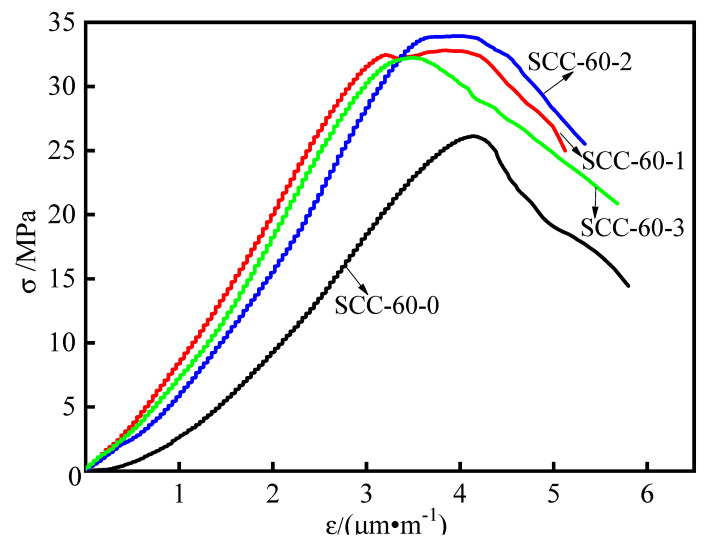
Stress-strain curve under axial compression.

According to the research results in Section 3.2.1, the workability of steel-fiber-reinforced SCC containing fly ash decreases sharply when steel fibers content is greater than 0.75%. Such results are also reported in the literature [38]. Thus, the steel fiber volume fraction (0~0.75%) is selected in the axial compression deformation property test. Load-displacement data is collected from axial compression tests of different steel-fiber-reinforced SCCs containing a high level of fly ash, and then converted into stress and strain values by calculation of Equations (1) and (2). Corresponding stress-strain curves are shown in Figure 7.

(1) σ=F/A

(2) ε=ΔL/L

where F is axial load, A is section area, ΔL is axial compression displacement, and L is specimen height.

The strain energy of concrete is usually expressed by the area under the stress-strain curve, which represents the energy absorbed per unit volume when the concrete loses its bearing capacity. In this study, based on the area of the stress-strain curve at 1.15 times peak strain, the strain energy of steel-fiber-reinforced SCC containing a high level of fly ash under axial compression is analyzed.

(3) $$V_{\varepsilon }=V\int_{0}^{\varepsilon_{1}}\sigma d\varepsilon$$

here $V_{\varepsilon }$ is strain energy, V is volume of specimen, $\varepsilon_{1}$ is peak strain, σ is axial compressive stress, and ε is axial compressive stress.

In order to compare the deformation characteristics of different specimens under axial compression, the strain energy consumed per unit strength of unit volume concrete under ultimate stress is taken as the comparison parameter.

(4) $$\Gamma =\frac{V_{\varepsilon }}{\sigma_{0}V}$$

where Γ is relative toughness, and $\sigma_{0}$ is limit stress.

According to the Equations (3) and (4), and the stress-strain curve in Figure 7, the limit stress ($\sigma_{0}$), peak strain ($\varepsilon_{0}$), strain energy ($V_{\varepsilon }$), and relative toughness (Γ) are calculated and listed in Table 7.

It is clear that the stress-strain curves of steel-fiber-reinforced SCC with 60% fly ash reach a peak, and then the plastic deformation stage appears. In the axial compression deformation test, it is seen that the failure of all specimens is diagonal crack failure, and the failure surface is connected by steel fibers. The formation process of these cracks is similar. Under the action of axial pressure, micro-cracks begin to appear on surface of specimen, and then these micro-cracks rapidly expand and penetrate to form macro-cracks in the unstable period of crack propagation, until the specimen is destroyed. According to the data in Table 7, the introduction of steel fibers provides a distinct increase for the $\sigma_{0}$ compared to the specimens without steel fibers, but when the percentage is from 0.25% to 0.75%, the changing amplitude of the $\sigma_{0}$ is not obvious, which has the same explanation as the above-mentioned influence of steel fibers on compressive strength. However, the $\varepsilon_{0}$ has a gradually decreasing trend as the percentage of steel fibers changes. This may be because the introduction of steel fiber brings more original micro-defects into the concrete matrix. In the process of compression, steel fibers with high elastic modulus have an important role in small crack opening, and with the expansion of the crack opening, the steel fiber cannot play its crack resistance effect in the larger crack opening, resulting in the reduction of $\varepsilon_{0}$ in macro [39].

The calculation results of the parameters in Table 7 reflect that when the percentage of steel fibers is 0.25%, the $V_{\varepsilon }$ and Γ of SCC are highest, reaching 90.14 N·m and 0.90 × 10^−3^, respectively. They increase by 47% and 15%, respectively, compared to specimens without steel fiber. Nevertheless, as the steel fibers exceed 0.25%, the $V_{\varepsilon }$ and Γ decrease, since there are more original defects in the SCC. The reason may be that the introduction of steel fiber is very detrimental to the propagation and penetration of large-scale cracks, increasing the crack propagation path and prolonging the cracking time of the specimens. Thus, more energy is absorbed when specimens are damaged, resulting in an increase of the $V_{\varepsilon }$ and Γ. However, once the volume fraction of steel fibers exceeds a critical value, the “scaffolding” effect between steel fibers reduces its fluidity. There are more original defects in the interface transition zone between the steel fibers and cement paste, resulting in the decrease of the $V_{\varepsilon }$ and Γ to a certain extent.

## 4. Conclusions

In this paper, SCC containing high levels of fly ash (50%, 60%, and 70%) was prepared to discuss its workability in fresh state and mechanical properties under hardened state. Then, an experimental program of steel-fiber-reinforced SCC with 60% fly ash was conducted to present the influence of steel fibers volume fraction on the workability, mechanical properties, and axial compression property. Based on the experimental results, the following conclusions can be drawn:

(1) Compared with SCC prepared with pure cement, the workability of SCC containing a high level of fly ash is generally improved, while there is a negative correlation between mechanical properties and fly ash content.
(2) The workability of the. mixture is negatively correlated with the volume fraction of the steel fibers. When the percentage of steel fibers is greater than 0.75%, workability decreases sharply. This shows that 0.75% volume fraction is a critical value of workability for steel-fiber-reinforced SCC with a high level of fly ash.
(3) The study concluded that the compressive strength of steel-fiber-reinforced SCC containing a high level of fly ash does not change significantly, but its flexural-tensile properties are dramatically improved by steel fibers. Incorporating 1.00% steel fibers into SCC containing 60% fly ash can make its splitting tensile strength and flexural strength increase by 22% and 58%, respectively.
(4) In terms of axial compression deformation characteristics of steel-fiber-reinforced SCC containing 60% fly ash, the parameters have been increased by adding steel fibers, especially the $V_{\varepsilon }=V\int_{0}^{\varepsilon_{1}}\sigma d\varepsilon$ and $\Gamma =\frac{V_{\varepsilon }}{\sigma_{0}V}$. The incorporation of 0.25% steel fibers is optimal for the $V_{\varepsilon }$ and Γ under axial compression deformation.

## Figures and Tables

**Table 1:** Properties of ordinary Portland cement.

Setting Time/Min		Compressive Strength/MPa		Flexural Strength/MPa	
initial	final	3d	28d	3d	28d
267	342	32.5	52.5	6.4	8.7

**Table 2:** Main components of fly ash.

Components	SiO_2_	Al_2_O_3_	Fe_2_O_3_	CaO	K_2_O	MgO	Na_2_O	Loss
Content/%	54.84	24.73	6.04	4.07	1.72	0.72	0.12	4.52

**Table 3:** Property parameters of steel fiber.

Density/(g/cm^3^)	Length/mm	Aspect Ratio	Tensile Strength/MPa
7.8	35 ± 3	63	≥380

**Table 4:** Mix proportion of SCC containing high level fly ash.

Code	Cementitious Materials/(kg/m^3^)			W/C	Sand Proportion	Water Reducing Agent Content (by Mass)/(%)
	Cement	Fly Ash	Lime Powder			
SCC-0	479	-	-	0.35	0.50	0.5
SCC-50	237.5	208.0	33.5	0.35	0.50	0.4
SCC-60	191.6	241.6	45.8	0.35	0.50	0.4
SCC-70	141.5	275.0	62.5	0.35	0.50	0.4

**Table 5:** Influence of different fly ash content on workability of SCC.

Code	Filling Ability		Passing Ability		Separation Rate/%
	Slump Flow/mm	T_500_/s	J-Ring Flow/mm	D-Value/mm	
SCC-0	655	3.9	640	15	15.8
SCC-50	660	4.0	640	20	15.3
SCC-60	670	3.0	660	10	12.0
SCC-70	700	3.0	685	15	11.6

**Table 6:** Influence of steel fiber volume fraction ($\varphi_{f}$) on workability of SCC containing a high level of fly ash.

Code	$\varphi_{f}/\%$	Filling Ability		Passing Ability		Separation Rate/%
		Slump Flow/mm	T_500_/s	J-Ring Flow/mm	D-Value/mm	
SCC-60-0	0	670	3.0	660	10	12.0
SCC-60-1	0.25	660	5.1	620	40	8.3
SCC-60-2	0.50	620	9.1	585	35	8.1
SCC-60-3	0.75	590	10.3	550	40	8.5
SCC-60-4	1.00	560	14.8	460	100	6.3

**Table 7:** Parameters of the axial compression property of steel-fiber-reinforced SCC containing a high level of fly ash.

Code	$\varphi_{f}/\%$	$\sigma_{0}/\mathrm{MPa}$	$\varepsilon_{0}/(µm\cdot m^{-1})$	$V_{\varepsilon }/(N\cdot m)$	$\Gamma \times 10^{3}$
SCC-0	0	26.13	4.14	61.18	0.78
SCC-1	0.25	32.81	3.85	90.13	0.90
SCC-2	0.50	33.93	4.02	87.31	0.86
SCC-3	0.75	32.23	3.49	72.17	0.75

## Data Availability

All data are available within the article.
